# Supplementary material for: Ulcerative skin lesions among children in Cameroon: It is not always Yaws
Source: PLoS Negl Trop Dis. 2021 Feb 16;15(2):e0009180. doi: 10.1371/journal.pntd.0009180 (PMC7909670; doi:10.1371/journal.pntd.0009180)
Supplement: Attachment — Submitted filename: 20210116_reply to reviewers Plos NTD Yaws H ducryei.docx [file pntd.0009180.s002.docx]

We thank the editor and the reviewers for their thorough review of our manuscript and their constructive comments, which helped improve our manuscript. We provide below our answers to each comment and indicate how we have incorporated modifications into the revised manuscript.

Reviewer #2:

The objectives of this study are not clearly spelt out. While in lines 92-93 of the main text, the authors state that they intended to "better understand the aetiology of skin ulcers among children residing in yaws endemic regions in Cameroon," the same indicate in the abstract (lines 6 and 7) that they "investigated the presence of T. p. pertenue and H. ducreyi DNA in skin ulcers in children living in yaws-endemic regions in Cameroon." The latter is more specific and though narrower in its scope, is more applicable to the methodology described. The study design as described cannot address the stated objectives in lines 92-93 of the main text.

**Authors’ reply: We thank the reviewer for pointing this out.**

**We reworded lines 92-93 of the manuscript as follows to state more clearly the objectives of our work, lines 96-99 read as follow:**

**To better understand the role played by *T. p. pertenue* and *H. ducreyi* in the etiology of skin lesions among children residing in yaws endemic regions in Cameroon, we analysed skin lesion samples collected in the context of three yaws-like outbreaks among children in 2017 and 2019 using nucleic acid amplification tests targeting *T. p. pertenue* and *H. ducreyi***.

The authors should be conscious of the broad aetiology of skin ulcers in tropical/sub-tropical areas which include Yaws but also other common differentials such as tropical ulcers of mixed aetiology and other bacterial lesions such as ecthyma.
Given the understanding that this study was performed within the context of an outbreak investigation, for which reason ethical clearance was not sought, a lot more will be expected of the authors to clearly describe the aetiology of the ulcers encountered by the Public Health Surveillance actors.

**Authors’ reply:**

**A more detailed description of the agents involved in skin ulcers was added to the manuscript. See lines 90-95.**

Going with the broad objective(s) identified within the main text, the authors have not also incorporated the full process of clinical decision making involving some element of clinical history (e.g. evolution of ulcers) and examination (e.g. categorization of ulcers based on appearance - superficial, deep, sloping edge, etc) in their text - these should be included graphically or in tabular forms within manuscript. The educated reader will want to know more about the types of ulcers being tested; while a gold standard diagnostic test greatly supports clinical decision making, the former on its own does not replace the value of good clinical decision making which can have up to an 80% yield from just clinical history.

Also, and consequently, the paper is largely silent on the aetiology of skin-ulcers not attributable to H ducreyi and or T. p. pertenue and the influence of trauma which is highlighted by the authors.

**Authors’ reply:**

**In addition to our two previous replies as mentioned above:**

**Children were examined clinically for presenting yaws-like lesions (papilloma, papules, hyperkeratosis and ulcers) and the location of the lesions on the body (See Table 2). All the lesions occurred spontaneously within 6 weeks of the onset of the outbreak. The lesions most of the time began as a small eruption on the skin before evolving to a papule and then to a papilloma and/or ulcer within 2-3weeks. There were no notions of trauma.**

Some thoughtful questions to be considered by authors:
- If only one ulcer per individual was sampled even in the presence of multiple ulcers, which the authors state may have reduced the probability of detecting T. p. pertenue and hence the disparity between reactive serological results and PCR confirmation, what was done to resolve this disparity?

**Authors’ reply:**

**We noted in the discussion that the sampling procedure might be responsible for the discrepancy between reactive serological results and PCR confirmation. Future studies will include samples from multiple lesions to try and resolve this disparity.**

Of ethical concern too is what was done with those study subjects in question?

**Authors’ reply:**

**We respectfully do not see any reason for concern regarding the ethical conduct of the study and the management of the patients enrolled. Yaws was suspected based on clinical examination and was confirmed based on a stepwise RDT testing with RDT and DPP serology as recommended by WHO for a sample of the children seen with yaws-like lesions during the outbreak. After obtaining positive yaws serology in some of the subjects we concluded the outbreak to be yaws and therefore treated every subject with a clinical yaws-like lesion and their household contacts with a single dose of Azithromycin, according to the WHO recommended guidelines for “Total Targeted Treatment” of yaws. (Eradication of yaws: a guide for programme managers. Geneva: World Health Organization; 2018. (WHO/CDS/NTD/IDM/2018.01) Licence: CC BY-NC-SA 3.0 IGO.)**

In the case of outbreak 1, 35 individuals neither had H. Ducreyi or T. p. pertenue confirmed in lesions - what then did they have, and what happened to them? Although that may argued as being in the context of the National Surveillance, it is relevant to make the conclusions and recommendations from this paper stronger.

**Authors’ reply:**

**During the outbreak response, after having confirmed it as yaws with the aid of serological rapid diagnostics tests (RDT & DPP), all individuals who presented with yaws-like lesions, whether confirmed by serology or not and their house contacts were treated with a single dose of azithromycin tablets. Samples collected from those with lesions were analyzed by PCR long afterwards when the technology became available at the reference lab in Yaounde.**

In lines 205-208, the authors make statements about the differences in age distribution and gender distribution for both sets of PCR confirmed cases of interest, which are not supported within text. The authors should show provide more details about the statistical analysis and results (probably within the table where they present the results).

**Authors’ reply:**

**Additional details about the statistical analysis were provided as requested. Rather than within the table, we chose to update the Materials section of the manuscript. See lines 170-174.**

Reviewer #3: Line 124, The beginning of the sentence should start with a capital letter. Correct is ‘If positive’.

**Authors’ reply:**

**This mistake was corrected.**

Line 139, A period is required at the end of the sentence.

**Authors’ reply:**

**This mistake was corrected.**

Line 302-305, I agree that the emergence of azithromycin resistance mutations is very important in the countermeasures of T.p. pertenue. But why aren't your samples tested for resistance mutations?
I recommend you to detect resistance mutations of azithromycin in your sample.

**Authors’ reply:**

**We agree that checking for azithromycin-resistance mutations would be useful and would add importance to our work, given the threat that resistance poses to yaws eradication efforts.**

**However, we wish to remind the reviewer that the work reported here was part of a difficult capacity-building project that required years. This time was needed to enable our laboratory to perform molecular analysis of these samples due to the necessity to provide training to personnel and obtaining the appropriate reagents to perform the assays.**

**We currently plan on implementing the additional assays necessary to evaluate azithromycin resistance in these samples as well as those that will be collected prospectively.**

**However, for the time being, we are not able to foresee when this capability will be available in our lab. We believe however, that also without these additional data, our work is worth publishing.**

Reviewer #2: The results are clearly presented within the manuscript, but the details of statistical analysis as indicated in previous section, should be added.

**Authors’ reply:**

**Additional details about the statistical analysis were provided as requested. Rather than within the table, we chose to update the Materials section of the manuscript. See lines 170-174.**

A suggestion is also made to the authors, to add a clearer map detailing where cases were found within the Lomie, Bikoe and Bipindi districts.

**Authors’ reply:**

**We added a more detailed map, Figure 1.**

Reviewer #3: Line 225-227, These data were not found in the figures or tables. Where are these shown?

**Authors’ reply:**

**We included the data in Table 6.**

Line 227, It was written that “T. pallidum but not T. p. pertenue was detected (from skin lesion samples of a child)”, which is very important. Because there are not so many reports of Nonvenereal Transmission of Syphilis, (J Williams et. al, Indian J Sex Transm Dis. 1990;11(1):27-8 and Pingyu Zhou et. al, Sexually Transmittsd Diseases 2009;36(4):216-217, etc.) you should discuss this case. Also, this case may be venereal, as the report by Elise Klouman et al. suggests (Genitourin Med 1997;73:522-527). In addition, Bejel, another nonvenereal syphilis, has been reported in warm, moist areas, which is not the arid areas, unlike traditional wisdom, so this point may also need to be considered.

**Authors’ reply:**

**We might have caused an involuntary misunderstanding; therefore we wish to specify that the 2 samples we are referring to had detectable *Treponema pallidum* DNA, but for which we could not confirm the subspecies using the *tprL* PCR. As this reviewer certainly knows, *Treponema pallidum* DNA is detected using qPCR, whereas the subspecies *pertenue* is identified based on the size of the *tprL* amplicon obtained by qualitative PCR. Both techniques have a different lower limit of detection, with the latter being less sensitive. Consequently, samples with a low concentration of *Treponema pallidum* DNA may be missed when *tprL* gene is targeted by PCR.**

**It is well known that the treponemal and non-treponemal tests do not differentiate among subspecies, but based on the clinical history of the patient, we believe, based on the location of the lesions (legs) and the age of the children (10 and 11 years), to be unlikely that these 2 cases (for which we could not amplify the tprL target) were actual syphilis lesions obtained through non sexual transmission.**

**We cannot exclude that the two children were infected with *T. p*. subspecies *endemicum*, as both bejel and yaws are transmitted by skin-to skin contact and affect similar demographic groups. However, bejel was never previously reported in Cameroun. Also the localization of the skin lesions does not support the diagnosis of bejel. Ulcers associated with bejel are usually located in the oral cavity or nasopharynx. Whereas our two patients had skin lesions on the lower limb and trunk, which is more typical of yaws infection.**

**We added this argumentation to the discussion section lines: 300-315.**

--------------------

Reviewer #2: The conclusions of this study are of public health relevance and support similar evidence from the Solomon Islands and Ghana, as indicated by the authors. While the data presented by study adds to the body of knowledge supporting the need for better characterization and diagnosis of skin ulcers in tropical/sub-tropical areas, addressing the issues about the methodology will further enhance its advancement of our understanding of this topic. Clearly there are still several questions unanswered, even from their own clearly spelt out limitations of the analysis.

The statement in lines 279 to 281 - "Our results also illustrate how difficult it is to clinically diagnose yaws in skin ulcers" may be right but the subsequent statement "and that molecular assays should be used to determine the aetiology of the lesion" may be a logical jump that is not supported by the data or the analysis, given the limitations spelt out and the challenges regarding the methodology to answer the broad objective identified in lines 92-93.

**Authors’ reply:**

**We agree and changed our statement as follows “Our results also illustrate how difficult it is to clinically diagnose yaws in skin ulcers. In order to improve the accuracy of the diagnosis of yaws, molecular assays should be used for the detection of *T. p. pertenue* in yaws-like lesions. “ (Lines 296-299)**

**We also reworded the objectives of our study, cfr lines 96-99.**

While the authors' call for a continuous surveillance program after MDA's is justified, again the data given the limitations and challenges with methodology, do not support restricting the nature to just the use of molecular diagnostic tools. It makes sense from their data presented, that post MDA, surveillance should involve both serological and molecular methods, fine-tuned to meet operational constraints.

**Authors’ reply:**

**We agree with the reviewer that post MDA surveillance using serology may reduce costs and will be easier to implement, however we argue that in the broad context of Yaws eradication, molecular diagnostic and resistance detection tools are more appropriate.**

**Serology may remain positive even after successful treatment: the presence of Treponemal IgG is mostly livelong and non-treponemal antibodies may decrease slowly. Another drawback of serology is its cross reactivity with other treponemal infections such as bejel and syphilis, which may co-exist with yaws infections.**

**Nowadays we mainly rely on real time PCR methods for what concerns the molecular diagnosis and resistance detection. However, molecular methods which are more compliant with the ASSURE criteria are being designed or are under validation. In a very near future these new molecular diagnostic tools may be employed in surveillance activities. (Becherer, L., Knauf, S., Marks, M., Lueert, S et al. (2020). Multiplex mediator displacement loop-mediated isothermal amplification for detection of Treponema pallidum and Haemophilus ducreyi. Emerging Infectious Diseases, 26(2), 282–288. https://doi.org/10.3201/eid2602.190505)**

Reviewer #3: Line 234, A period is required at the end of the sentence.

**Authors’ reply:**

**This oversight was corrected.**

Line 302-305, I agree that the emergence of azithromycin resistance mutations is very important in the countermeasures of T.p. pertenue. But why aren't your samples tested for resistance mutations? (Reposting of Methods comment.)

**Authors’ reply:**

**We agree that checking for azithromycin-resistance mutations would be useful and would add importance to our work, given the threat that resistance poses to yaws eradication efforts.**

**However, we wish to remind the reviewer that the work reported here was part of a difficult capacity-building project that required years. This time was needed to enable our laboratory to perform molecular analysis of these samples due to the necessity to provide training to personnel and obtaining the appropriate reagents to perform the assays.**

**We currently plan on implementing the additional assays necessary to evaluate azithromycin resistance in these samples as well as those that will be collected prospectively.**

**However, for the time being, we are not able to foresee when this capability will be available in our lab. We believe however, that also without these additional data, our work is worth publishing.**

Line 296, Citation [29,30] should be [28,29].

**Authors’ reply:**

**We apologize for the oversight. We updated the references and changed the citation order accordingly.**

Line 301, Citation [28] should be [30].

**Authors’ reply:**

**We apologize for the oversight. We updated the references and changed the citation order accordingly.**

Line 306, Citation [29] should be [31]

**Authors’ reply:**

**We apologize for the oversight. We updated the references and changed the citation order accordingly.**

--------------------

Reviewer #2: Statements in lines 63-64 and line 68 should be supported by in-text citations.

**Authors’ reply:**

**We added in-text citations to the end of the statements contained in lines 63-64 and 68.**

Reviewer #3: (No Response)

--------------------


Reviewer #1: Line 84 - I think it is clear that H Ducreyi definitely causes skin ulcers so I would remove the word possible here. Equally in the discussion I think  there is considerably less doubt about its causative role than is presented here - it is isolated from lesions, clearly causes ulcers in an experimental model, the lesions heal in response to targeted treatment of ducreyi. We find staph aureus on intact skin but it is also clear that it definitely causes skin lesions so I am not sure the fact that we find ducreyi on intact skin really alters the overall strong evidence that kochs postulates have been fufilled.

**Authors’ reply:**

**The word “possible” was removed, and we reworded the discussion to more clearly reflect that both pathogens are etiological agents of ulcers**

1) The information on sero-diagnosis should be presented much much earlier in the results as this is key.
For example given that 0 cases in outbreak 2 & 3 were associated with positive serology the fact that these cases was not yaws is completely unsurprising.

- Serology breakdown should be included in the tables related to the outbreaks.

**Authors’ reply:**

**The serology results were moved higher up and are now presented after the section summarizing the study population.**

**Serology data related to the outbreaks were presented in a new Table 3.**

2) Related to this Figure 2 is difficult to follow.

I would suggest a table showing
Percent dual positive (Trep/non-Trep), percent trep only, percent both negative - and then the PCR breakdown across these groups.

**Authors’ reply:**

**Figure 2 was replaced by Table 6.**

3) I presume all the lesions were ulcerative without papillomas? Ulcers have a hugely wide differential and it is not at all surprising that most are not caused by T.p.pertenue (simialr results for example are found when people investigate possible Buruli ulcers)

**Authors’ reply:**

**Data on lesion appearance was added to Table 1 whenever available.**

4) No information is given about what reporting in Cameroon is based on but I presume as with elsewhere that it is clinical reporting without any serological confirmation. If so then it should be made clear that national reporting data is likely to be highly unreliable.

**Authors’ reply:**

**The reported Yaws cases are based on first clinically observation of suspected cases which are then confirmed based on a stepwise RDT testing with RDT and DPP serology as recommended by WHO for a sample of the children seen with yaws-like lesions during an outbreak. If RDTs are not available standard syphilis serology is recommended. After obtaining positive yaws serology in some of the subjects it is concluded that the outbreak is due to be yaws.**

5) Lines 293 - I would suggest referencing the syphilis literature as it is very clear (and unsurprising) that primary lesions may be PCR positive before seroconversion and therefore before a DPP or similar assay will be positive.

**Authors’ reply:**

**Appropriate literature was referenced.**

6) The raw data required to reproduce the analysis are not available with the submission. PLease provide these.

**Authors’ reply:**

**Raw data are now provided as supplemental material.**

Reviewer #2: Overall a useful paper which is well written, but will greatly benefit from a review of the methodology to meets its broad objective and enhance its value in furthering our knowledge on the aetiology of skin-ulcers in tropical/sub-tropical areas. While the authors indicate that ethical clearance was not sought since this was in the context of the national skin ulcer surveillance and no supplementary data was collected, given that this secondary data analysis identifies people in living specific districts (they may be of a particular tribe for example), the issues of potential harm to subjects and return for consent need to be considered by an ethical review board, even if it is not a complete review. This will strengthen this publication and the conclusions from it.

**Authors’ reply:**

**A more detailed descriptions of the agents involved in ulcers was added to the manuscript. See lines 90-95.**

**We complemented the ethical clearance section and it reads now:**

**“We did not seek additional ethical clearance as the analysis were done in the context of the national skin ulcer lesion surveillance approved by the National Committee on Research Ethics for Human Health (approval reference 2016/08/800/CE/CNERSH/SP). No supplementary data were collected. All parents/ guardians provided oral consent and voluntary opted into the sampling of their children for surveillance purposes. All individuals were managed and treated according to the national Cameroonian guidelines and in line with the WHO recommended guidelines for “Total Targeted Treatment” of yaws [24].”**

Reviewer #3: Line 306, Macrolide Resistance of T.p. pertenue was described in 2015 by David Šmajs et. aｌ. in Am. J. Trop. Med. Hyg., 93(4):678-683. So, citation should be changed.

**Authors’ reply:**

**We apologize for the oversight and changed the citation accordingly.**
